# Transcutaneous electric nerve stimulation (TENS) in dentistry- A review

**DOI:** 10.4317/jced.51586

**Published:** 2014-12-01

**Authors:** Vikrant Kasat, Aditi Gupta, Ruchi Ladda, Mitesh Kathariya, Harish Saluja, Anjum-Ara Farooqui

**Affiliations:** 1 MDS, Reader. Department of Oral Medicine and Radiology, Rural Dental College, Loni; 2 BDS, Private Practioner. Delhi; 3 MDS, Sr. Lecturer. Department of Prosthodontics, Rural Dental College, Loni; 4 MDS, Reader. Department of Pedodontics, Rural Dental College, Loni; 5 MDS, Reader. Department of Oral and Maxillofacial Surgery, Rural Dental College, Loni; 6 MDS, Sr. Lecturer. Department of Oral Medicine and Radiology, Rural Dental College, Loni

## Abstract

Transcutaneous electric nerve stimulation (TENS) is a non-pharmacological method which is widely used by medical and paramedical professionals for the management of acute and chronic pain in a variety of conditions. Similarly, it can be utilized for the management of pain during various dental procedures as well as pain due to various conditions affecting maxillofacial region. This review aims to provide an insight into clinical research evidence available for the analgesic and non analgesic uses of TENS in pediatric as well as adult patients related to the field of dentistry. Also, an attempt is made to briefly discuss history of therapeutic electricity, mechanism of action of TENS, components of TENs equipment, types, techniques of administration, advantages and contradictions of TENS. With this we hope to raise awareness among dental fraternity regarding its dental applications thereby increasing its use in dentistry.

** Key words:**Dentistry, pain, TENS.

## Introduction

**Figure 1:**
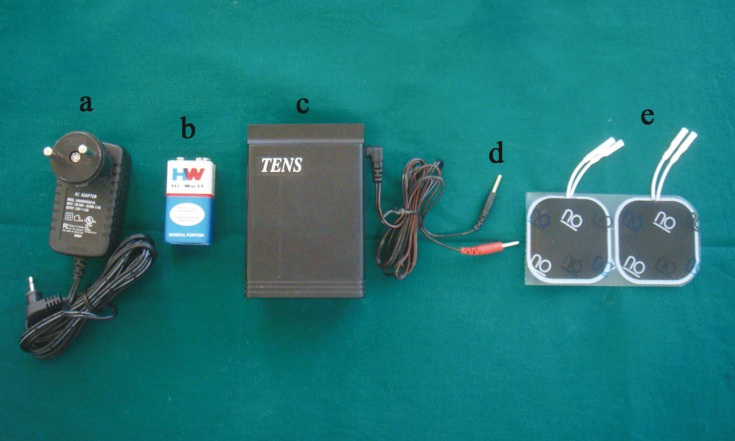
a) AC adapter; b) 9V battery; c) TENS unit; d) Lead wires; e)Electrodes.

Pain has been a constant tormentor of mankind since time immemorial. Techniques used to control pain are broadly divided into pharmacological and non pharmacological methods. Most common pharmacological means to curb pain in dentistry is the use of local anesthesia during dental procedures and analgesics for the postoperative pain. Use of local anesthesia instills fear in a many patients as it requires the use of the ‘horrifying’ syringe. A non-pharmacological method for pain control is the use of transcutaneous electrical nerve stimulation [TENS] (1). FDA [Food and Drug Administration] has approved TENS as a method of pain alleviation (2) and classified it as class II device in 1972. During TENS therapy, pulsed electrical current is generated either by A.C. mains (Fig. 1) or using batteries [usually 9V] (Fig. 1) and delivered across the intact skin surface via electrodes to stimulate superficial nerves for localized pain relief (3). TENS is commonly used by health professionals for acute and chronic pain management (4,5). In dentistry, though TENS has potential applications, it is not used that frequently. Hence, the purpose of this article is to review its applications in dentistry so as to raise awareness among dental fraternity regarding its dental applications. For review a search of “PubMed” was made with the keywords “TENS AND dentistry,” “TENS AND trigeminal neuralgia,” “TENS AND orofacial pain,” “electronic dental anaesthesia.” Also, after searching references of full text articles, relevant articles were included. For review, articles published in English language with no time limit were selected.

## Brief history

Electricity has been used for alleviation of pain since the era of ancient Greeks, Romans and Egyptians who used live Torpedo marmorata [electric ray], a type of electric fish for pain relief. In modern era, John Wesley in 18th century introduced electrotherapy for the relief of pain from sciatica, headache, kidney stone, gout, and angina pectoris. Use of electricity for relief of dental pain was first described in 19th century by a physician named Francis. In 20th century, various dental handpieces that provided an electrical current to the tooth via the bur were used to relieve pain during cavity preparation. After a lot of research, TENS or electronic dental anesthesia as it is called in dentistry has established itself as an anesthetic agent (6).

## Mechanism of action

Analgesic effect of TENS is based on two main theories- Gate control theory of pain and endogenous opiod theory.

## Gate control theory of pain

Gate control theory of pain proposed by Melzack and Wall (7) in 1965, is the most popular theory to explain the mechanism of action of TENS. They suggested that substantia gelatinosa present in the dorsal horn of spinal cord functions as a gate control system that modulates the afferent patterns from peripheral fibers before they influence the first central transmission [T] cells of spinal cord. Small unmyelinated ‘C’ fibers transmit pain and their activity keeps the gate in relatively open position. Activity of large myelinated A fibers exert presynaptic inhibition on input from C fibres and is responsible for closure of gate, thus preventing impulses from reaching T cells (7). Pain control can be achieved by increasing large fiber input and decreasing small fiber input and thus, closing the gate.

## The endogenous opioid theory

In 1969, Reynolds (8) showed that electrical stimulation of periaqueductal gray region of the midbrain produces analgesia equivalent to that induced by morphine. Subsequently, this led to the discovery of several morphine like chemicals called endorphins which exist at various levels of the pain control pathway. Thus, alternative explanation for the mechanism of action of TENS is that it stimulates the release of endogenous opioids in the spinal cord which could result from activation of local circuits within the spinal cord or from activation of descending pain-inhibitory pathways (9).

## Classification of TENS

Clinically, TENS is applied at varying frequencies, intensities, and pulse durations of stimulation. Depending upon frequency of stimulation, TENS is broadly classified into 2 categories: [1] High frequency TENS [>50Hz]. [2] Low frequency TENS [<10 Hz] (9-11). High frequency TENS operates via the gates theory producing only short term analgesia, whereas low frequency TENS operates through release of endogenous opioids which causes a more systemic and long-term response (9-11).

- Tens Equipment

Main parts of TENS system are: [1] TENS unit. [2] Lead wires. [3] Electrodes

- TENS unit

It is an electric pulse generator. It has two variations: [1] “Clinical” model- This is used by dentist in the clinic and is connected to the buildings electrical outlet to generate power (2). [2] “Patient” model- This is small and portable unit which can be carried in a pocket by the patient or can be secured to the belt or clothing of the patient. It contains battery as a power source (Fig. 1).

- Lead Wire

These connect electrodes to TENS unit to establish electrical connection (Fig. 1).

- Electrodes

By means of electrodes, electric flow from TENS unit is converted into an ionic current flow in the living tissue. Electrodes can be placed extraorally or intraorally. Extraoral electrodes are of two types: [1] Carbon- impregnated silicone rubber electrodes- They are flexible and coupled to the skin surface through the use of electrically conductive gel. They are retained in place with surgical tape. [2] Tin plate or aluminum electrodes- These don’t conform to the body and are coupled to the skin surface with tap water retained within cotton pad or sponge.

The intraoral electrodes are cotton roll electrodes, clamp electrodes and adhesive electrodes. Adhesive electrodes are the most widely used type nowadays. These electrodes are thin and flexible so can adapt easily to the oral mucosa (6) (Fig. 1).

## TENS- Types and techniques

Three main types of TENS are described in the literature – 1. Conventional TENS 2.

Acupuncture-like TENS [AL-TENS] and 3. Intense TENS. Different TENS techniques are used to selectively activate different afferent nerve fibers (4).

1. Conventional TENS

`It is the most commonly used method for delivering currents in clinical practice. It uses high frequency [between 10-200 pulses per second [pps]], low intensity [amplitude] pulsed currents to activate the large diameter Aβ fibers without concurrently activating small diameter Aβ and C [pain-related] fibres or muscle efferents (6). It produces segmental analgesia which has a rapid onset [< 30 min after switch-on] and offset [< 30 min after switch-off]. Generally, conventional TENS can be administered regularly throughout the day, but intermittent breaks should be taken to reduce the skin irritation (4). In conventional TENS pulse delivery is usually continuous, but same effect can also be achieved by delivering the pulses in ‘bursts’ or ‘trains’ which has been labeled as pulsed or burst TENS by some authors.

2. Acupuncture-like TENS [AL-TENS]

It uses low frequency [less than 10pps, usually 2-4 pps], high intensity pulsed currents to activate the smaller diameter Aδ fibers arising from muscles [ergoreceptors] by the induction of phasic muscle twitches. It produces extrasegmental analgesia which has a delayed onset [> 30 min after switch-on] and offset [>1 h after switch-off]. AL-TENS can be used for about 30 minutes at a time as fatigue may develop with ongoing muscle contractions.

3. Intense TENS

It uses high frequency [upto 200 pps], high intensity pulsed currents which are just bearable to the patient. It activates small diameter Aδ cutaneous afferents and produces extrasegmental analgesia which has a rapid onset [< 30 min after switch-on] and delayed offset [>1 h after switch-off]. Intense TENS can be used for about 15 minutes at a time as the stimulation may be uncomfortable.

## Advantages

1. It is non-invasive, safe (12) and can be used to achieve anesthesia in needle-phobic patients (5).

2. As compared to local anesthesia there is no postoperative anesthesia after the TENS unit is turned off (5).

3. Patients are able to self-administer TENS treatment and learn to titrate dosages accordingly to manage their painful condition. This results in positive acceptance by the patients (5).

## Contraindications

1. Apprehensive patients- usage of TENS requires patient co-operation, hence the procedure shouldn’t be at-tempted in patients with a communication handicap or a mental disability.

2. Patients with cardiac pacemakers-(6,13). If the electrode placement is in the thoracic area, TENS currents can interfere with the function of pacemaker except fixed rate pacemaker. Since the patients are generally unaware of the kind of pacemaker that they use, it is advised not to use TENS in these patients.

3. Patients with cerebrovascular problems- patients with a history of aneurysm, stroke and transient ischaemia shouldn’t be treated using TENS, as it stimulates peripheral blood flow which can be fatal in such cases (6).

4. Epileptic patients- TENS “pulses” have the potential to trigger a seizure (6).

5. Pregnant patients- As such there are no specific side effects. However, since there has been no FDA approval, the usage is frowned upon (6).

6. Acute pain cases/pain of unknown etiology- usage of TENS in undiagnosed cases may hinder in the diagnosis (6).

## Applications in dentistry

Apart from its analgesic effect, TENS can also be used to produce non-analgesic physiological effects and has been found to be beneficial in the management of xerostomia. Various applications of TENS in dentistry are summarized below

1. Dental treatment in pediatric patients

A commonly observed negative behavior in pediatric patients is fear towards syringes. Use of TENS has positive effects on the behavior of pediatric patient which in turn decreases the anxiety levels as it removes the “fear of needle”. Studies have shown that 53 -78% children prefer TENS over local anesthesia (14-16). In pediatric patients, TENS has been used effectively to control pain during various procedures like pit and fissure sealant placement, cavity preparations, minor extractions and endodontic procedures.

Abdulhameed *et al.* (17) in 1989 evaluated effect of TENS on tooth pain threshold in 30 children who required pit and fissure sealants in first or second permanent molars. They observed 33% increase in tooth pain threshold after 8 minutes of TENS application. Also, decrease in stress-associated tachycardia was evident by pulse oximeter.

teDuits *et al.* (15) in 1993 conducted a study in 27 children in whom two antimere teeth needed restorations. They randomly used TENS on one side and traditional local anesthesia on the other side at the same appointment. Though they found no significant difference in pain perception [regarding dentin sensitivity and rubber dam clamp replacement] between the two treatment modalities on Eland Color Scale, majority [78%] of the patients preferred TENS over local anesthesia. Oztaş *et al.* (16) in 1997 conducted a similar study for restorations in primary molars in children aged 7 to 9 years and observed that 56% of the patients preferred TENS over local anesthesia.

Harvey and Elliott (18) in 1995 found that TENS is effective in pain reduction during cavity preparations in pediatric patients. In a double blind study, out of 20 patients requiring class 1 amalgam restorations in mandibular permanent 1st molars, they treated 10 patients using TENS and 10 patients without using TENS. Visual analogue scale [VAS] and ANOVA test revealed a significant decrease in pain readings in the TENS group patients compared to the control group.

Baghdadi (14) in 1999 conducted a study in 28 children to determine the effectiveness of TENS in comparison with local anesthesia for restorative procedures. Each child had 2 symmetric teeth requiring class I cavity preparations. Both teeth were restored in the same appointment, one using TENS and other using local anesthesia. He used color scale and the sound, eye, and motor scale for pain assessment and North Carolina Behavior Rating Scale for behavior assessment. He found no significant difference between the two methods, but 53.6% of the patients preferred TENS.

Munshi *et al.* (19) in 2000 used TENS for the management of pain during treatment procedures such as minor extractions, restorations, and pulp therapy in 40 children between the ages of 5-12 years. They found significantly favorable results with TENS.

Dhindsa *et al.* (4) in 2011 compared efficacy of TENS with 2% lignocaine in reducing the pain during extraction, cavity preparation, pulpotomy, and pulpectomy of deciduous teeth in 180 pediatric patients. Response to pain and comfort and effectiveness of anesthesia were compared using the VAS, verbal pain scale [VPS] and Lickert scale. ANOVA values using TENS and 2% lignocaine showed no significant difference [*P*>0.05]. They concluded that TENS can be a useful adjunct in pediatric patients during various minor dental procedures.

2. Dental treatment in adult patients

In adults TENS has been used successfully as an excellent analgesia during various procedures like rubber dam placement, cavity preparation, pulp capping and other endodontic procedures, prosthetic tooth preparations, oral prophylaxis as well as extractions. It is also used to reduce the discomfort from injection of local anesthesia and to alleviate periodontal pain associated with orthodontic separation.

Roth and Thrash (20) in 1986 used TENS to assess its effect on periodontal pain associated with orthodontic separators placed mesial and distal to the upper first molars in 45 adult patients. Patients on TENS reported significant decrease in pain on VAS at the 24, 36, 48 hour assessment periods, whereas the control group experienced post adjustment pain even after a period of 60 hours.

Malamed *et al.* (5) in 1989 reported success rate of TENS in 109 patients requiring class I, II, III, IV, or V restorations. They found that TENS was more successful in anterior than posterior teeth and was more effective for shallow [85.8% success rate] and moderate cavity [85.5%] than deep cavity i.e. more than 2 mm into dentine [59.5%].

William Stenberg (21) in 1994 reported use of TENS to control pain during cavity preparations in a 24 year old malignant hyperthermia susceptible patient and found favorable results. Earlier amide type of local anesthetics was thought to be hazardous in these patients, but now studies have proved safety of local anesthetics. If still physician or patient expresses fear to local anesthetics, then TENS can be used.

Yap and Ho (12) in 1996 did a clinical comparison of the efficacy, as perceived by 10 clinicians and 30 patients, of electric and local anesthesia for operative procedures based on a 5-point Lickert scale. Local anesthesia was perceived to be significantly more effective by both evaluator groups, yet a staggering 93.3% of the patients preferred TENS.

Quanstrom and Libed (22) in 1994 compared the ability of TENS and topical anesthesia in controlling the pain from injection of local anesthesia. Two maxillary infiltration injections were performed on 21 patients, one after electric anesthesia and the other after topical anesthesia. They found that less pain was experienced by patients after use of TENS and therefore patient’s preferred electrical anesthesia over topical anesthesia in the ratio of 3:1.

Meechan *et al.* (23) in 1998 conducted a study in 100 adult patients to compare the use of 2% lignocaine and TENS as means of reducing the discomfort of inferior alveolar nerve block injections. They found that the use of TENS reduces injection discomfort during inferior alveolar nerve block anesthesia as compared to use of 2% lignocaine.

According to Hochman (24), TENS is less successful in “skeptical” and “highly pain-sensitive” patients. He reported 83% success rate of TENS for soft-tissue procedures like oral prophylaxis, 76% for restorative procedures and 55% for crown and bridge work.

TENS has also been used in combination with nitrous oxide-oxygen or diazepam to achieve analgesia during dental treatment. Quanstrom and Milgrom (25) in 1989 combined TENS with nitrous oxide-oxygen in 309 patients and compared it with TENS alone in 62 patients to check efficacy of pain control during restorative procedures without using local anesthetic. They found successful outcome in 84% of TENS combination and 55% of TENS alone cases. They found that “patients fear” is single most important factor in preventing effective use of TENS and factors like depth of cavity preparation or group of tooth are not significant. Varrese and Guerrini (26) combined TENS with diazepam for performing various dental procedures over a period of 9 years from 1980-1989. They found that sufficient analgesia was obtained to carry out procedures like extractions, devitalizations, prosthetic preparations, third molar surgery, and enucleation of tooth buds.

3. In chronic pain of maxillofacial region

TENS has been used successfully to alleviate chronic pain of TMJ syndrome, trigeminal neuralgia, and post herpetic neuralgia.

- In TMJ syndrome

Katch (2) reported use of TENS to control pain of TMJ syndrome in a 10-year-old girl and achieved 50-75% of pain relief. Apart from the three treatment cycles of 20 minutes each, patient used TENS along with ice massage at home to maintain pain control.

- In trigeminal neuralgia

Singla *et al.* (27) conducted a study on 30 patients with trigeminal neuralgia who were given continuous bursts of current for 20 minutes daily for 20-40 days over the path of the affected nerve with a portable TENS machine. Patients were subsequently evaluated at 1 and 3 months intervals for pain by VAS, VPS and functional outcome scale which showed significant decrease in pain.

Yameen *et al.* (28) used TENS to treat trigeminal neuralgia pain in 31 patients who were refractory or partially responsive to drug therapy. Severity of pain was assessed on a VAS prior to and 15 days after treatment. They found that 83.7% patients improved significantly with application of TENS and a constant mode gave slightly better therapeutic results than burst mode of TENS.

Thorsen and Lumsden (29) reported an interesting case of trigeminal neuralgia in a 36-yr-old man that showed immediate and long-term remission of symptoms when intense discharge of TENS was delivered accidentally. Hence, they thought that TENS at an intense level may result in long-lasting effects.

- In post-herpetic neuralgia

In post-herpetic neuralgia most of the larger myelinated afferent nerve fibers are destroyed and therefore, normal presynaptic inhibition of inputs of C fibers does not occur (7). This is responsible for the pain and abnormal sensitivity of the skin seen in post-herpetic neuralgia. The rationale for using TENS is that it would reintroduce the normal inhibition by increasing the activity of remaining large fibers (30).

Nathan and Wall (30) in 1974 used TENS to relive pain of severe post-herpetic neuralgia in 30 patients in whom all other forms of therapy had failed. Patients treated themselves by a battery operated TENS apparatus [frequency 15 - 180 Hz] for 12 hours or more. Out of these 30 patients, good results were seen in 11 patients. They found that patients with the most severe pain do not get relief from TENS probably because of insufficient large myelinated fibres present to produce the inhibition.

Mittal *et al.* (31) in1998 used TENS in 10 patients suffering from pain of post-herpetic neuralgia. TENS therapy was given daily at 70 Hz frequency for 20 minutes for a total of 10 days. TENS therapy was successful in achieving 50% or more reduction in pain in 60% of patients. They found that patients with a shorter duration of post-herpetic neuralgia responded better to TENS therapy.

4. In acute orofacial pain

Hansson and Ekblom (32) studied effect of high frequencies [100Hz], low frequencies [2Hz] and placebo TENS for relief of acute orofacial pain in 62 patients who had suffered pain for 1-4 days. A decrease in pain intensity exceeding 50% was found in 38% of patients receiving either form of TENS, where only 10% of patients recei-ving placebo TENS experienced a pain reduction of more than 50%.

5. In patients with xerostomia

Application of TENS increases the salivary flow rate in healthy individuals as well as in xerostomic patients. Hargitai *et al.* (33) in 2005 found increased salivary flow in two-thirds of healthy adult subjects after application of TENS on the skin overlying the parotid glands. Their results also suggested that for TENS to be effective, baseline saliva flow should be present.

Pattipati *et al.* (34) in 2013 used electrostimulation in 90 healthy adults and found that application of TENS over parotid region results in increased salivary flow rate.

Weiss *et al.* (35) in 1986 conducted a randomized, double-blind, placebo-controlled trial in patients with xerostomia to study the effects of electrostimulation on salivary flow. For this, they used an intraoral device where the probes contacted the tongue and the palate. They found improvement in approximately 75% patients, but their assessment method was very subjective.

Steller *et al.* (36) in 1988 conducted a double-blind study in 29 patients of Sjögren’s syndrome to determine whether an electrical stimulus applied to the tongue and hard palate by a battery-operated device could stimulate salivary flow. The device was used three times a day for three minutes each and continued for a period of four weeks. They concluded that electrical stimulation was useful only in those patients with some residual salivary flow present.

Talal *et al.* (37) in 1992 conducted a multi-center double-blind study in patients of Sjögren’s syndrome to evaluate the ability of an electro-stimulator device to increase the production of saliva. Out of 77 Sjögren’s syndrome patients, 40 were assigned to active devices, 37 were assigned to placebo devices and treatment was continued for a period of 4 weeks. They found a statistically greater increase in the production of saliva in patients using active devices than placebo patients.

Wong *et al.* (38) in 2003 conducted a single institutional Phase I-II study to assess the effectiveness of AL-TENS device [Codetron] for relief of dry mouth in 46 patients with radiation-induced xerostomia. Residual salivary function was present in all recruited patients. Codetron treatment of acupuncture points preselected according to traditional Chinese medicine principles was given over a period of 12 weeks with 2-week break after 6 weeks of treatment. The results of the study indicated that this treatment method improved whole saliva production and the effects were sustained for at least 6 months after treatment completion.

Wong *et al.* (39) in 2012 conducted a similar study to assess feasibility of AL-TENS device [Codetron ™] delivery in a multicenter setting and its efficacy in reducing radiation-induced xerostomia in 48 patients with radiation-induced xerostomia. AL-TENS was done for 20 minutes two times a week for 12 weeks. They concluded that it is feasible to use the device in multicentre setting as they got 94% patient compliance. Also, a positive treatment response was noted in 86% of patients.

## Conclusion

In conclusion, though TENS can’t replace local anesthesia, it can be used for pain relief during various dental procedures. Its analgesic and non analgesic physiologic effect can be used in the management of a variety of conditions affecting maxillofacial region.
